# Mitochondrial genomes of two subspecies of *Citellophilus tesquorum*: Insights into the molecular evolution and phylogenetic analysis within Siphonaptera

**DOI:** 10.3389/fvets.2026.1785674

**Published:** 2026-04-29

**Authors:** Mingna Duan, Yuran Xia, Dandan Jiang, Shaobo Tang, Rui Hou, Lanling Tian, Tao Wang, Tianguang Ren, Xing Yang

**Affiliations:** 1 Integrated Laboratory of Pathogenic Biology, College of Preclinical Medicine, Dali University, Dali, Yunnan, China; 2 Northwestern Polytech Univ, Sci & Technol Thermostructural Composite Mat Lab, Xi'an, Shaanxi, China; 3 Northwestern Polytech Univ, Ningbo Inst, Ningbo, Zhejiang, China; 4 School of Public Health, Dali University, Dali, Yunnan, China; 5 School of Government Administration, Baoshan University, Baoshan, Yunnan, China

**Keywords:** *Citellophilus tesquorum*, comparative analysis, evolution, mitochondrial genome, phylogeny, plague

## Abstract

**Background:**

*Citellophilus tesquorum* is a major flea vector in natural plague foci across the Palaearctic region of China, representing a significant threat to veterinary and public health. Precise identification and sound taxonomic delimitation of its subspecies are critical for effective vector surveillance and evolutionary research.

**Methods:**

We first performed morphological identification to guarantee specimen authenticity and accuracy, then sequenced and annotated the complete mitochondrial genomes of *C. t. dzetysuensis* and *C. t. mongolicus* using the Illumina NovaSeq platform. Comparative mitogenomic and phylogenetic analyses were conducted with 33 publicly available flea mitochondrial genomes to explore genomic evolution, codon usage patterns, and phylogenetic relationships.

**Results:**

The two newly sequenced mitogenomes showed high structural conservation and a strong AT bias, consistent with typical features in Siphonaptera. Natural selection was identified as the dominant force driving codon usage bias. Phylogenetic reconstructions revealed that *C. t. dzetysuensis* clustered strongly with *C. t. sungaris*, while *C. t. mongolicus* formed a well-supported sister clade, indicating genetic divergence among these subspecies.

**Discussion:**

The observed genetic differentiation is likely driven by long-term geographic isolation and host adaptation. This study establishes an integrative morphological and mitogenomic framework, enriches flea mitochondrial genomic resources, and provides key molecular evidence for the phylogeny, taxonomy, and surveillance of flea vectors.

## 1 Introduction

The order Siphonaptera, commonly referred to as fleas, encompasses small, wingless, jumping parasitic insects that undergo complete metamorphosis. These insects predominantly infest mammals and birds, with a considerable number of species being specifically adapted to rodents (1). According to the most recent revision of the taxonomy, there are currently 19 recognized families within this order, 259 genera (comprising 252 extant genera and 7 fossil genera), 2,695 valid extant species (including subspecies), and 17 fossil species (2). Most fleas are significant ectoparasites of both domestic and wild animals. While many fleas associated with domestic animal primarily cause irritation through biting, they can also induce flea-allergic dermatitis and anemia in susceptible hosts. Certain flea species may embed their mouthparts or even entire bodies into mammalian or avian tissues, leading to localized inflammation and various pathological lesions. Moreover, some fleas act as intermediate hosts for trypanosomes that infect domestic animals, and several species are capable of transmitting pathogens-including viruses and trypanosomes-to their hosts (3). Fleas are also recognized vectors of numerous zoonotic diseases. They transmit *Rickettsia typhi*, responsible for murine typhus; *Rickettsia felis*, associated with flea-borne spotted fever; *Bartonella henselae*, which causes cat-scratch disease; *Bartonella quintana*, the pathogen behind trench fever; and *Yersinia pestis*, the causative agent of plague (4, 5). Consequently, fleas represent not only important agents of parasite disease in veterinary medicine affecting livestock, poultry, and companion animals, but also significant vectors of zoonoses at the public health level, posing serious threats to animal production efficiency, pet health, and human health safety.

*Citellophilus tesquorum* Wagner, 1898 serves as a significant and active vector for *Y. pestis* across the extensive Palearctic region (6). This species parasitizes several ground squirrels species of the genus *Spermophilus*, predominantly in Southern Europe, Central Asia, the Caucasus, South Siberia, Mongolia, and Northern China (7). At the subspecific level, *C. tesquorum* is classified into nine recognized subspecies (8). Among these, four subspecies are distributed in China: *C. t. altaicus*, *C. t. dzetysuensis*, *C. t. mongolicus*, and *C. t. sungaris* (9). Previous studies have detected spotted fever group rickettsiae (SFGR) and *Coxiella burnetii* in *C. t. dzetysuensis* (10, 11), which has also been found to harbor *Bartonella* and *Trypanosoma* species (12). Accurate identification of flea species and systematic taxonomic research are essential for the effective prevention and control of flea-borne diseases. Currently, flea classification and identification primarily rely on morphological characteristics. However, traditional morphological methods present notable limitations, including minimal morphological differentiation among subspecies, susceptibility to environmental and sexual variation, high subjectivity, and an inability to identify cryptic species (13). Recent molecular systematic studies of *C. tesquorum* have predominantly utilized DNA sequence data from a limited number of genetic markers in nuclear DNA (particularly the internal transcribed spacer region of rDNA) and mitochondrial DNA (*cox1* gene) (8). Nevertheless, the resolution provided by these individual molecular markers appears insufficient for robust phylogenetic inference. Mitochondrial genomes are increasingly recognized as powerful tools for resolving phylogenetic relationships due to their ability to generate high-resolution genomic data that surpass traditional morphological and single-gene molecular markers (14, 15). Mitochondrial genomics has been employed to investigate the phylogeny of the order Siphonaptera; however, its utility remains limited by the paucity of mitochondrial genomic data for many taxa. As of now, the NCBI database contains mitogenome sequences for over 30 flea species, yet taxonomic representation remains inadequate.

This study sets three primary objectives: (1) To conduct, for the first time, sequencing and analysis of two important vector flea species endemic to plague foci in China—*C. t. dzetysuensis* and *C. t. mongolicus*—including detailed morphological descriptions and systematic analyses of their mitochondrial genome structure, nucleotide composition, and structural characteristics; (2) To integrate the newly sequenced genomes with 33 previously published flea mitochondrial genome sequences retrieved from the NCBI database, and perform comparative mitogenomic analyses, focusing on assessing the conservation of gene order, base composition bias, nucleotide diversity, evolutionary rates, and codon usage preferences; (3) To reconstruct phylogenetic relationships among taxa within the order Siphonaptera using concatenated nucleotide sequences of 13 mitochondrial protein-coding genes from 35 flea species. The purpose of this study is to enrich the mitochondrial genome data resources of fleas, provide basic data support for taxonomic research and phylogenetic relationship exploration of Siphonaptera.

## 2 Materials and methods

### 2.1 Specimen collection and morphological identification

In July 2024, a total of 41 adult fleas-comprising 27 females and 14 males-of C. t. dzetysuensis were collected via direct combing from the body surface of *Spermophilus undulatus* in Jinghe County, Xinjiang Uygur Autonomous Region, China (44°62′N, 82°89′E). In October 2024, 32 adult fleas-19 females and 13 males-of *C. t. mongolicus* were similarly collected from *Spermophilus dauricus* in Zhengxiangbai Banner, Inner Mongolia Autonomous Region, China (42°28′N, 114°98′E). All specimens were immediately preserved in 95% ethanol and subsequently transported to the laboratory for taxonomic identification. Morphological identification was performed by the experts in Siphonaptera taxonomy using qualitative comparative morphology under an Olympus stereomicroscope, strictly adhering to the diagnostic criteria outlined in *Fauna Sinica Insect Siphonaptera* (2nd edition, 2007) (9). Representative individuals exhibiting diagnostic morphological characters were permanently slide-mounted and digitally imaged using a Zeiss Axio Imager A2 microscope equipped with an integrated imaging system. Accurate morphological identification ensures that only valid target specimens were used for DNA extraction and sequencing, which is critical to avoid misidentification and ensure the authenticity of subsequent molecular data. This step links morphological evidence with mitochondrial genome analysis and supports the integrative taxonomic purpose of this study.

Following morphological confirmation, 12 adult specimens per subspecies were randomly selected for molecular analysis. Genomic DNA was extracted from individual fleas using the DNeasy Blood & Tissue Kit (QIAGEN, Hilden, Germany), following the manufacturer’s standard protocol. This study was conducted in strict accordance with institutional animal ethics guidelines and received formal approval from the Animal Ethics Committee of Dali University (Approval No. 2023-P2-280).

### 2.2 Mitogenomes sequencing, assembly, and annotation

PE libraries with 400 bp insects were constructed using the whole-gene shotgun approach and sequenced with 2 × 150 bp paired-end reads on an Illumina NovaSeq platform. The raw sequencing data underwent quality control filtering to generate high-quality clean data. Subsequently, *de novo* assembly of the clean data was performed using SPAdes v3.15.4 (16). The assembled contigs were compared against the NCBI nt database using blastn to identify mitochondrial sequences. Bandage v0.8.1^1^ was employed to visualize the connectivity between mitochondrial segments, while pilon v1.18 (17) was utilized to refine the assembly and correct potential errors, thereby obtaining the final mitochondrial genome sequence. Functional annotation of mitogenome was conducted using MITOS web server^2^ (18), and CGView was applied to generate the circle map of mitogenome (19).

### 2.3 Mitochondrial genome comparative analysis

In this study, the complete mitochondrial genomes of two vector flea species were sequenced and systematically compared with those of 33 additional flea species retrieved from GenBank (Table 1). Gene order and rearrangement were analyzed by comparing the annotated mitogenomes with published flea mitochondrial genomes, gene structure comparisons among different taxa were carried out using PhyloSuite v1.2.3 (20). The nucleotide composition of each gene was analyzed using DNAstar v11.1, and base skews were calculated using the formulas AT-skew = (A − T)/(A + T) and GC-skew = (G − C)/(G + C) (21). Nucleotide diversity (Pi) and non-synonymous (Ka)/synonymous (Ks) substitution rates for the 13 PCGs were evaluated through sliding window analysis (window size = 100 bp, step size = 25 bp) conducted in DnaSP v6.12.3 (22).

**Table 1:** The taxonomic information and GenBank accession numbers of the mitochondrial genomes from 35 Siphonaptera species were selected for comparative analysis and phylogenetic reconstruction.

Family	Genus	Species	Length (bp)					Accession No.
			Complete genome	PCGs	tRNAs	rrnL	rrnS	
Ceratophyllidae	*Ceratophyllus*	*Ceratophyllus wui*	18,081	11,057	1,432	1,239	780	NC_040301
Ceratophyllidae	*Monopsyllus*	*Monopsyllus anisus*	15,875	11,137	1,433	1,218	779	NC_073017
Ceratophyllidae	*Citellophilus*	*Citellophilus tesquorum sungaris*	15,345	11,030	1,429	1,248	781	PP418872
Ceratophyllidae	*Citellophilus*	*Citellophilus tesquorum dzetysuensis*	16,458	11,138	1,429	1,295	780	PV693698
Ceratophyllidae	*Citellophilus*	*Citellophilus tesquorum mongolicus*	15,373	11,042	1,431	1,296	780	PV693696
Ceratophyllidae	*Jellisonia*	*Jellisonia amadoi*	17,031	11,119	1,431	1,293	787	NC_022710
Ceratophyllidae	*Macrostylophora*	*Macrostylophora euteles*	16,027	11,114	1,431	1,287	780	OR774969
Ceratophyllidae	*Nosopsyllus*	*Nosopsyllus laeviceps*	16,533	11,143	1,433	1,196	774	PP838812
Ceratophyllidae	*Amphalius*	*Amphalius spirataenius*	14,825	11,135	1,435	1,299	779	OR855715
Leptopsyllidae	*Frontopsylla*	*Frontopsylla diqingensis*	16,153	11,125	1,437	1,304	789	PP083946
Leptopsyllidae	*Paradoxopsyllus*	*Paradoxopsyllus custodis*	15,375	11,111	1,432	1,290	780	OQ627398
Leptopsyllidae	*Frontopsylla*	*Frontopsylla spadix*	15,085	11,144	1,439	1,281	786	NC_073018
Leptopsyllidae	*Leptopsylla*	*Leptopsylla segnis*	15,785	11,138	1,420	1,274	780	NC_072691
Leptopsyllidae	*Amphipsylla*	*Amphipsylla qinghaiensis*	15,579	11,135	1,429	1,303	779	PQ571081
Ischnopsyllidae	*Thaumapsylla*	*Thaumapsylla breviceps orientalis*	15,631	11,147	1,434	1,296	787	PP973737
Ctenophthalmidae	*Ctenophthalmus*	*Ctenophthalmus yunnanus*	15,801	11,118	1,415	1,251	780	NC_085277
Ctenophthalmidae	*Ctenophthalmus*	*Ctenophthalmus quadratus*	15,938	11,126	1,405	1,250	783	NC_072692
Ctenophthalmidae	*Stenischia*	*Stenischia montanis yunlongensis*	15,651	11,118	1,425	1,281	784	OR780663
Ctenophthalmidae	*Stenischia*	*Stenischia montanis*	15,889	11,124	1,425	1,281	784	PP990561
Ctenophthalmidae	*Stenischia*	*Stenischia humilis*	15,617	11,118	1,424	1,266	785	NC_073020
Ctenophthalmidae	*Stenoponia*	*Stenoponia polyspina*	14,933	11,124	1,405	1,299	782	OR834393
Ctenophthalmidae	*Neopsylla*	*Neopsylla specialis*	16,820	11,142	1,408	1,262	791	NC_073019
Ctenophthalmidae	*Neopsylla*	*Neopsylla hongyangensis*	15,832	11,144	1,419	1,296	791	PP133648
Hystrichopsyllidae	*Hystrichopsylla*	*Hystrichopsylla weida qinlingensis*	17,173	11,129	1,424	1,224	786	NC_042380
Pulicidae	*Pulex*	*Pulex irritans*	20,337	11,095	1,439	1,294	793	NC_063709
Pulicidae	*Ctenocephalides*	*Ctenocephalides orientis*	22,189	11,082	1,425	1,303	799	NC_073009
Pulicidae	*Ctenocephalides*	*Ctenocephalides felis felis*	20,911	11,094	1,415	1,302	785	MW420044
Pulicidae	*Ctenocephalides*	*Ctenocephalides felis felis*	15,418	11,084	1,420	1,245	786	MK941844
Pulicidae	*Ctenocephalides*	*Ctenocephalides canis*	15,609	11,082	1,412	1,300	798	NC_063710
Pulicidae	*Ctenocephalides*	*Ctenocephalides felis*	20,873	11,093	1,416	1,299	787	NC_049858
Pulicidae	*Xenopsylla*	*Xenopsylla cheopis*	18,902	11,064	1,426	1,312	797	MW310242
Tungidae	*Tunga*	*Tunga penetrans*	17,279	11,094	1,426	1,280	783	PV426769
Stivaliidae	*Aviostivalius*	*Aviostivalius klossi bispiniformis*	16,593	11,059	1,436	1,317	791	OR774970
Stivaliidae	*Aviostivalius*	*Aviostivalius klossi bispiniformis*	18,669	11,050	1,434	1,316	791	PP963728
Vermipsyllidae	*Dorcadia*	*Dorcadia ioffi*	16,785	11,134	1,436	1,302	782	NC_036066

### 2.4 Codon analysis

Relative synonymous codon usage (RSCU) was assessed using CodonW v1.4.2,^3^ and the results were visualized via heat maps generated in Origin v2021. RSCU is a quantitative measure utilized to evaluate the degree of preference for specific synonymous codons within a genetic sequence. An RSCU value of 1 indicates no bias in codon usage, meaning all synonymous codons are used equally. A value greater than 1 implies a higher than expected frequency of a particular codon relative to its synonymous alternatives, whereas a value less than 1 reflects a lower than expected usage frequency (23).

Codon usage bias was analyzed from multiple perspectives through ENC-plot, neutral plot, and parity rule 2 (PR2), thereby enabling the assessment of the influence of both mutation pressure and selection pressure on codon usage patterns. The ENC-plot was constructed by plotting the effective number of codons (ENC/Nc) as the dependent variable (ordinate) against GC3s as the independent variable (abscissa). The theoretical curve indicates that codon bias is solely attribute to mutational pressure, as described by the formula is Nc = 2 + s + {29/[s^2^ + (1 − s)^2^]}, where s represents the frequency of GC3s. Deviations of genes below the theoretical curve suggest that codon usage patterns are significantly influenced by selective pressures (24). In the neutrality plot, a two-dimensional scatter plot was generated using GC3s and GC12 as the horizontal and vertical coordinates, respectively. A strong correlation between GC12 and GC3s-indicated by data points aligning closely along the diagonal—suggests that the regression coefficient approaches 1. This implies minimal variation in base composition across the three codon positions, indicating that codon usage bias is primarily driven by mutational pressure. Conversely, if such a correlation is weak, it suggests that natural selection plays a predominant role in shaping codon usage preferences (25). PR2 analysis calculated A3s/(A3s + T3s) and G3s/(G3s + C3s) based on the respective values of A3s, T3s, G3s, and C3s, and examined the relationship between these ratios. When A = T and G = C, indicating that gene is located at the central point (0.5, 0.5), codon usage is considered to be influenced only by random mutation. Deviations from this equilibrium (A ≠ T and/or G ≠ C) indicate that both natural selection and mutation contribute to codon usage bias (26). Correspondence analysis (COA) is a multivariate statistical technique widely employed to examine variations in RSCU and the distribution of genes within a multidimensional space (27). It is employed to examine differences in codon usage patterns across species and to investigate the evolutionary trends and selective pressures influencing these species. The aforementioned results were visualized using R v4.5.1 (28).

### 2.5 Phylogenetic analysis

Substitution saturation at each codon position of the PCGs was tested using the Xia method in DAMBE v6.4.81 (29) to ensure the reliability of subsequent phylogenetic analyses. We selected the mitochondrial genome of 35 flea species (including two newly sequenced species) as the ingroup and used *Boreus elegans* (HQ696579) as outgroup for phylogenetic analysis (Table 1). The nucleotide sequences of 13 PCGs were extracted using PhyloSuite v1.2.3 (20). Sequence alignment was performed with MAFFT v7.505 (30), followed by filtering of ambiguously aligned fragments and gap sites using Gblocks 0.91b (31). Data concatenation was performed in PhyloSuite v1.2.3 to generate the PCGs dataset, and the optimal partitioning scheme and evolutionary model, based on the Bayesian Information Criterion (BIC), were determined using ModelFinder v2.2.0 (32), as specified in Table S1. Bayesian Inference (BI) analysis was conducted using MrBayes v3.2.7 (33) with a partitioned model, running for 1 million generations, discarding the first 25% of samples as burn-in, and ensuring convergence with ASDSF values less than 0.01. Maximum Likelihood (ML) analysis was performed using IQ-TREE v2.3.6 (34) with 1,000 bootstrap replicates. Finally, the resulting phylogenetic tree was visualized using ITOL v6.9.1 (35).

## 3 Results

### 3.1 Morphological features

**Figure 1:**
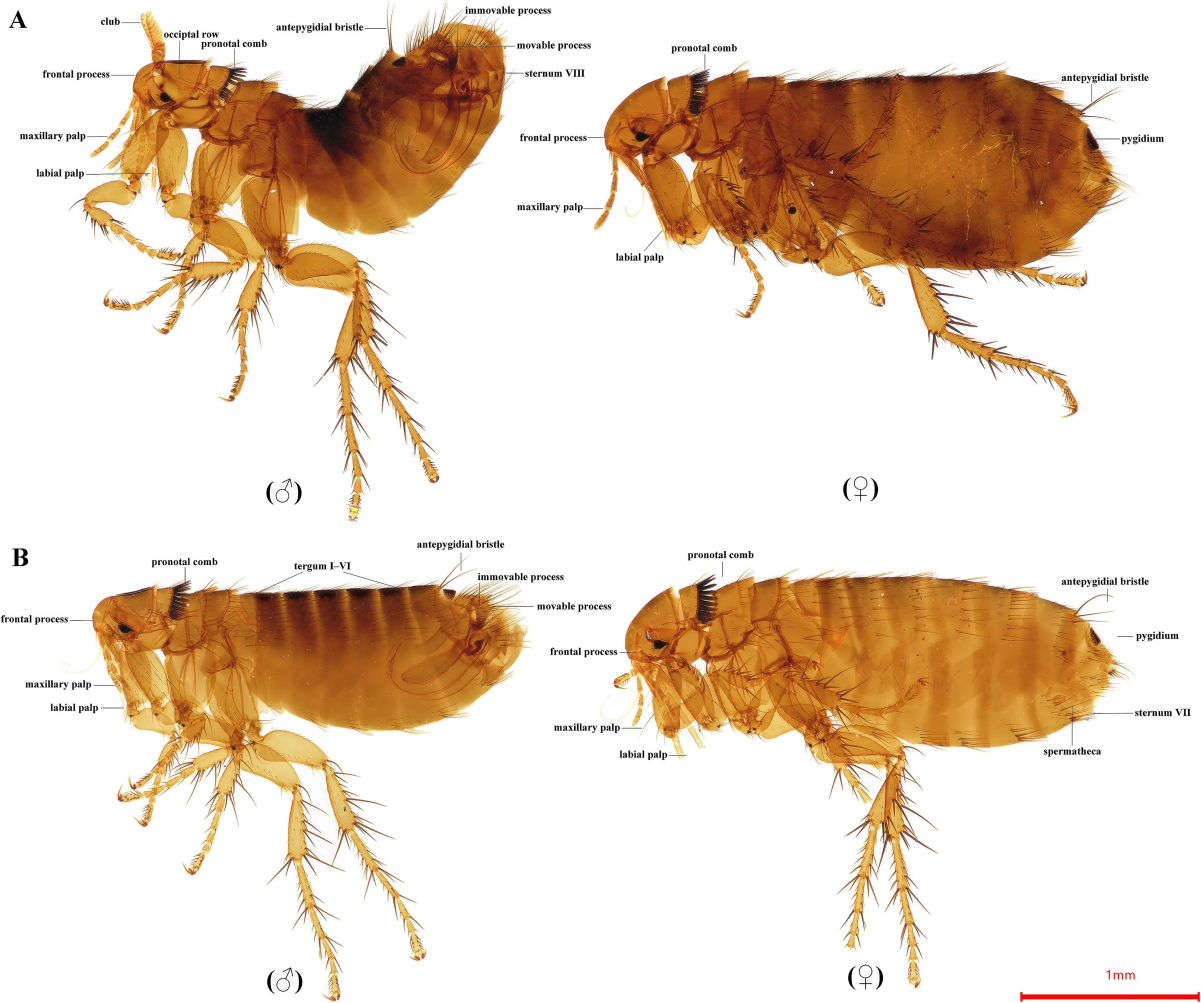
Morphological characteristics of *Citellophilus tesquorum dzetysuensis* **(A)** and *Citellophilus tesquorum mongolicus* **(B)**.

Morphological observation and identification were conducted to confirm the taxonomic status of *C. t. dzetysuensis* and *C. t. mongolicus* before DNA extraction and sequencing, which is a prerequisite to guarantee the accuracy and reliability of subsequent molecular experiments. Detailed morphological characteristics of the two subspecies were qualitatively compared and supplemented with descriptive statistics as follows:

For *C. t. dzetysuensis*, the frontal process is small and located at the lower 1/3 (male) to 1/4 (female) of the frontal margin. The eye row bears 3 bristles, and the occipital row has 1 bristle. The labial palp extends close to or slightly beyond the apex of the forecoxa. The pronotal comb consists of 18–22 spines, with the dorsal spines slightly shorter than the pronotum. The metanotum has 2–3 apical spinelets. Apical spinelets on abdominal terga I–V number 3–5, 4–7, 3–6, 1–5, and 0–2, respectively. In males, the apical membrane appendage of sternum VIII is reduced and lacks a fringe; the movable process is banana-shaped with a narrow apex and a broadly curved posterior angle; the posterior arm of sternum IX is wide and tapers apically; the aedeagal hook is highly reduced. In females, the posterior margin of sternum VII is broadly arched, usually with a dorsal or ventral sinus; the tail of the spermatheca is slightly longer than the head; the bursa copulatrix is weakly sclerotized (Figure 1A).

For *C. t. mongolicus*, the frontal process is dentate and located at the middle of the frontal margin. The labial palp is 5-segmented and extends below the trochanter. The pronotal comb has 14–21 spines, with dorsal spines approximately equal in length to the pronotum. Apical spinelets on abdominal terga I–VI number 2(3), 1–4, 1–3, 1(2), 0, and 0, respectively. In males, the apical membrane appendage of sternum VIII is dentate, sometimes with a fringe; the movable process is short, narrow-apical, and slightly axe-shaped; the aedeagal process is apex-capitate. In females, the posterior margin of sternum VII is highly variable; the head of the spermatheca is slightly shorter than the tail (Figure 1B).

### 3.2 Mitogenomes organization

**Figure 2:**
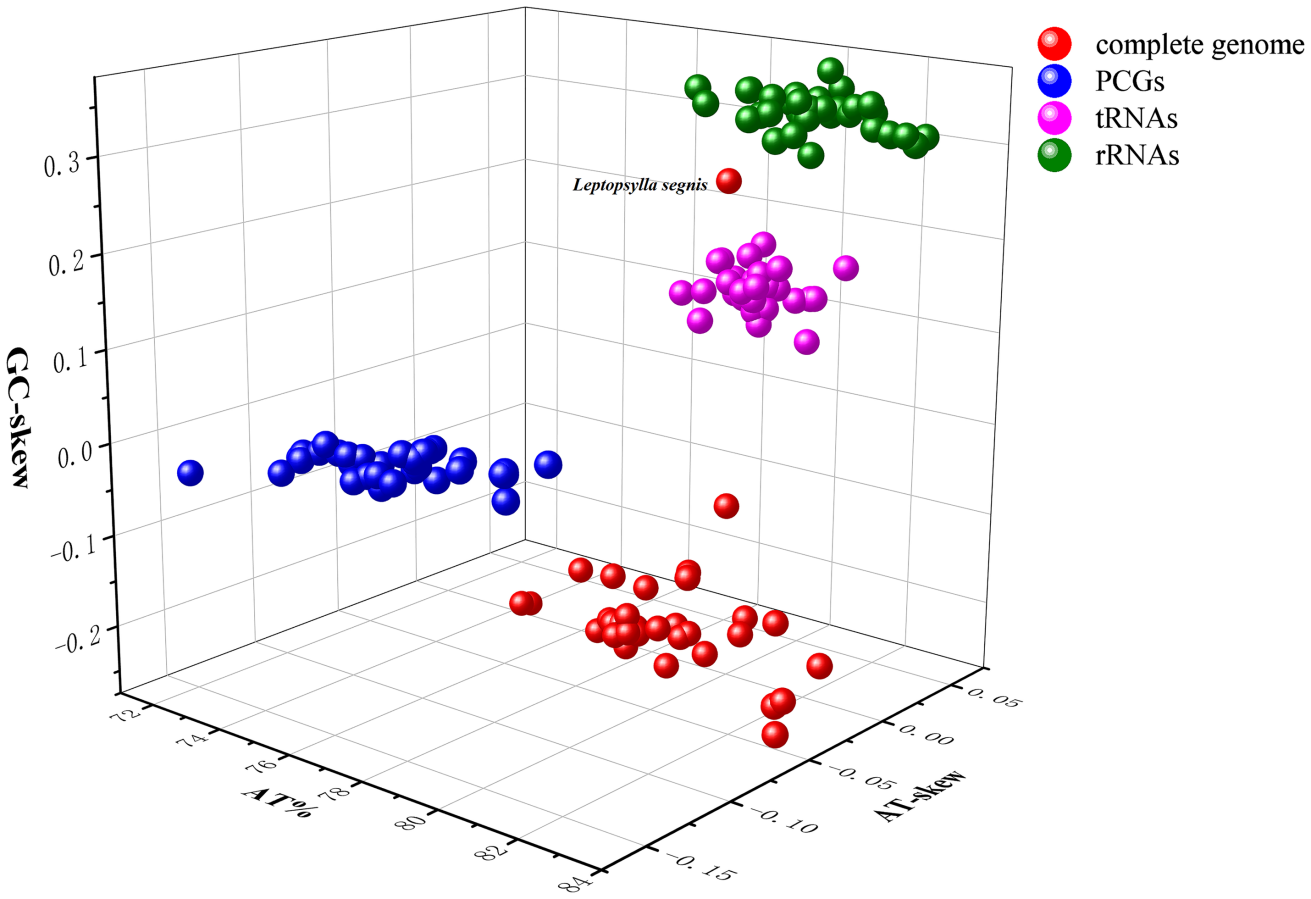
Three-dimensional scatter plot illustrating AT-skew, GC-skew, and AT% values derived from 35 mitochondrial genome sequences of Siphonaptera.

The mitochondrial genome lengths of 35 flea species ranged from 14,825 bp in *Amphalius spirataenius* to 22,189 bp in *Ctenocephalides orientis*. The mitogenomes of *C. t. dzetysuensis* and *C. t. mongolicus* were successfully sequenced, with length of 16,458 bp and 15,373 bp, respectively (Table 1). Both mitogenomes contain 13 PCGs, 22 tRNAs, and 2 rRNAs. Of these genes, 23 (including 9 PCGs and 14 tRNAs) are located on the heavy (H) strand, while the remaining 14 genes (including 4 PCGs, 8tRNAs, and 2 rRNAs) are found on the light (L) strand (Supplementary Table S2, Supplementary Figure S1). The sequence data have been deposited in the NCBI GenBank database under accession numbers PV693698 (*C. t. dzetysuensis*) and PV693696 (*C. t. mongolicus*). The adenine-thymidine (AT) content across the 35 mitochondrial genomes ranged from 74.6% in *Neopsylla hongyangensis* to 83.2% in *C. orientis*, demonstrating a notable AT bias. All analyzed mitogenomes exhibited AT-skew values between −0.051 and 0.024, and GC-skew values ranging from −0.268 to 0.248. The mitochondrial genome of *C. t. dzetysuensis* exhibited a high AT bias, with an overall AT content of 78.5%. Corresponding AT and GC skews were calculated as −0.031 and −0.220, respectively. In contrast, the mitogenome of *C. t. mongolicus* displayed a slightly higher AT content (78.3%), accompanied by AT and GC skews of −0.029 and −0.218, respectively. The majority of these values were negative, with only a few species showing positive skew, suggesting that the number of T and C nucleotides generally exceeds that of A and G nucleotides (Figure 2). In *C. t. dzetysuensis*, there were 13 spacer regions and 10 overlapping regions, where *C. t. mongolicus* exhibited 18 spacer regions and 7 overlapping regions. The length of the spacer regions in both species ranged from 1 to 62 bp, and the length of the overlapping regions ranged from 1 to 7 bp (Supplementary Table S2).

### 3.3 Protein-coding genes

Among the 35 mitochondrial genomes, the total length of the 13 PCGs ranged from 11,030 in *C. t. sungaris* to 11,147 bp in *Thaumapsylla breviceps orientalis* (Table 1). Of these 13 PCGs, 9 genes (*nad2*, *cox1*, *cox2*, *atp8*, *atp6*, *cox3*, *nad3*, *nad6*, and *cytb*) are located on the H strand, while the remaining 4 genes (*nad5*, *nad4*, *nad4l*, and *nad1*) are found on the L strand (Supplementary Table S2, Supplementary Figure S1). Among them, *nad5* is the longest gene, whereas *atp8* is the shortest. The total AT content across the 13 PCGs within the 35 mitogenomes varied between 72.3 and 80.3%. Furthermore, all mitogenomes exhibited negative AT skew, ranging from −0.156 to −0.117, while GC skew values fluctuated between −0.030 and 0.044. For the two newly sequenced subspecies—*C. t. dzetysuensis* and *C. t. mongolicus*—the AT contents of their PCGs were 75.8 and 76.6%, respectively. Corresponding AT skews were −0.148 and −0.144, while GC skews were 0.031 and 0.023, respectively (Figure 2).

A significant proportion of PCGs initiated with the standard start codon ATN, although TTG was utilized in the *atp8* and *atp6* genes. Additionally, non-standard start codons such as GCA, GTG, GTA, and AAA were identified in *cox1*. Most PCGs terminated with canonical stop codons TAA and TAG; however, some employed incomplete stop codons, namely T or TA (Supplementary Figure S2). These truncated stop codons are commonly found in mitogenomes of invertebrates and can be functionally completed through post-transcriptional polyadenylation (36).

**Figure 3:**
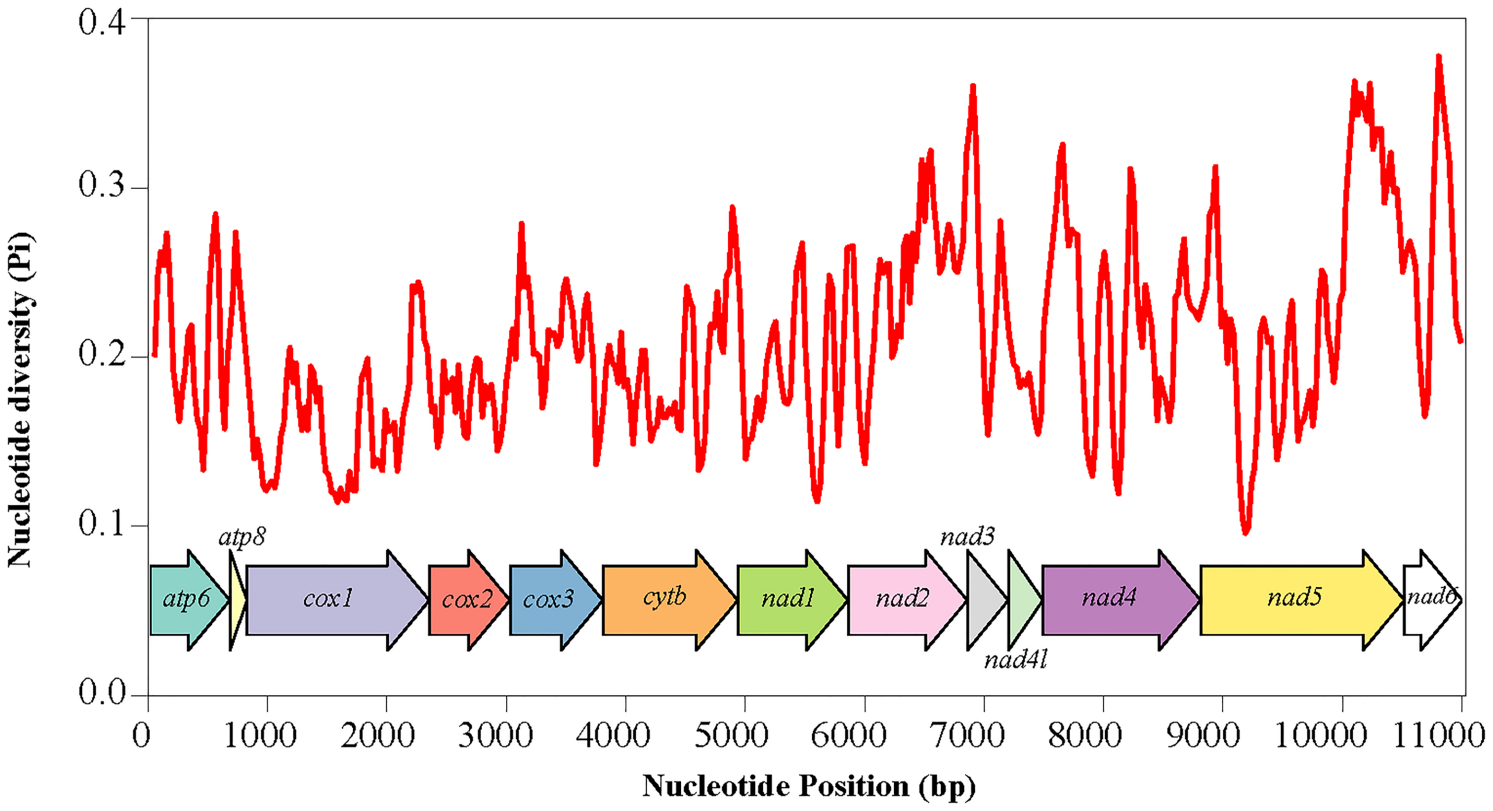
Estimates of nucleotide diversity (Pi) in the mitochondrial genomes of 35 species belonging to the order Siphonaptera.

**Figure 4:**
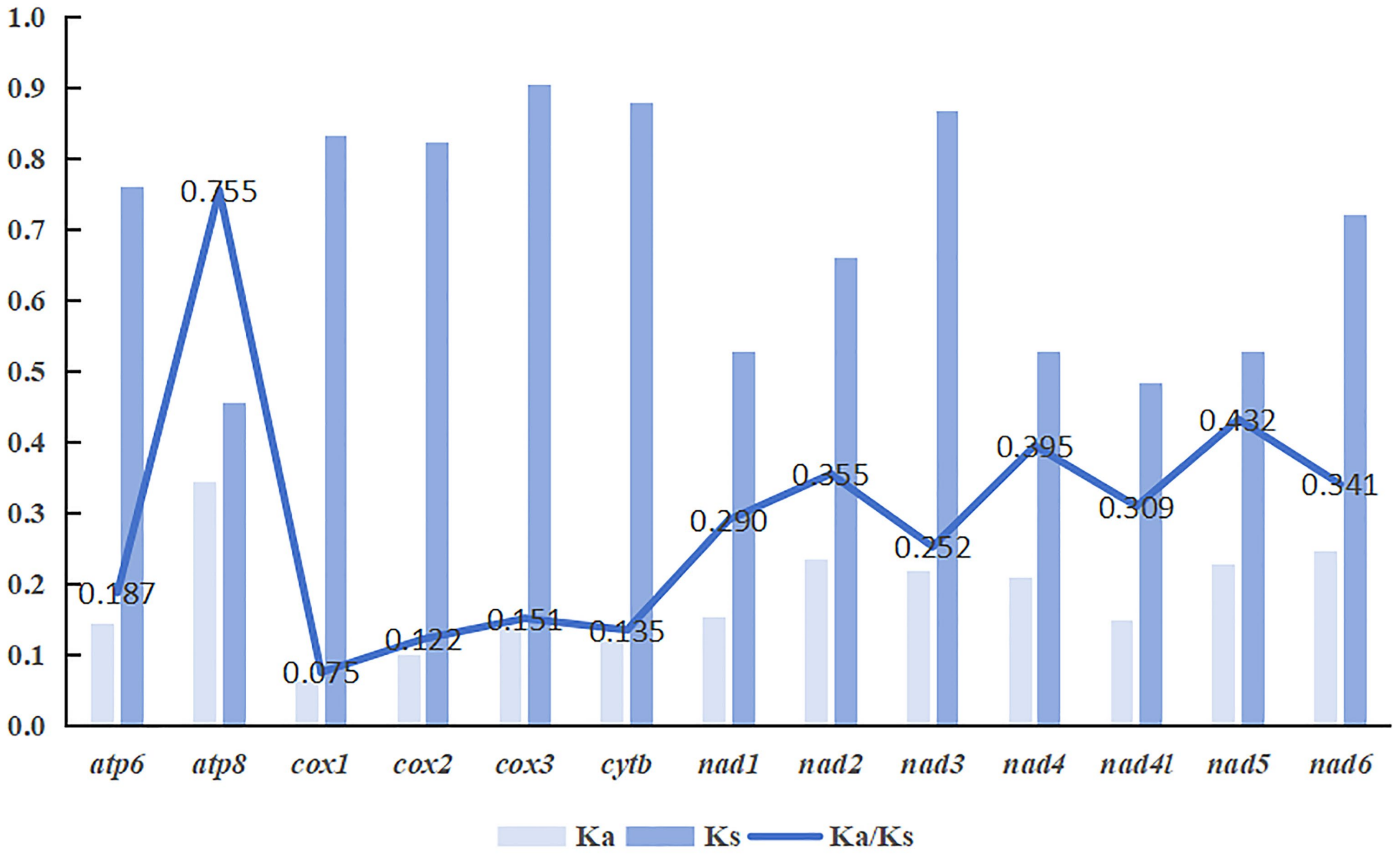
The evolutionary rates of 13 mitochondrial PCGs, represented by the ratio of nonsynonymous to synonymous substitutions (*K*a/*K*s), were analyzed across 35 flea species.

In this study, the nucleotide diversity and evolutionary rates of 13 PCGs in the mitogenomes of 35 Siphonaptera species were analyzed to elucidate their evolutionary patterns. The average Pi values ranged from 0.160 to 0.249, with *nad6* and *nad2* exhibiting the highest variability (0.249), followed by *nad3* (0.237) and *atp8* (0.236). In contrast, the *cox1* gene exhibited the lowest variability (0.160), indicating a high level of genetic stability of the *cox1* gene within fleas (Figure 3). The average Ka/Ks ratios across PCGs ranged from 0.075 to 0.755. The consistently low Ka/Ks values (<1) indicate that purifying selection predominantly shaped the evolution of all mitochondrial PCGs in the order Siphonaptera, making them suitable for phylogenetic analyses within this group. Among the genes analyzed, *atp8* exhibited the highest evolutionary rate with an average Ka/Ks value of 0.755, followed by *nad5* (0.432). In fleas, both the *atp8* gene and the NADH dehydrogenase complex genes demonstrated relatively high evolutionary rates, whereas *cox1* showed the lowest evolutionary rate (0.075) (Figure 4). The results from both Pi and Ka/Ks analyses suggest that the *cox1* gene evolves at a low pace and is therefore appropriate for constructing DNA barcoding databases to support traditional taxonomic identification of species.

### 3.4 Codon usage pattern analysis

**Figure 5:**
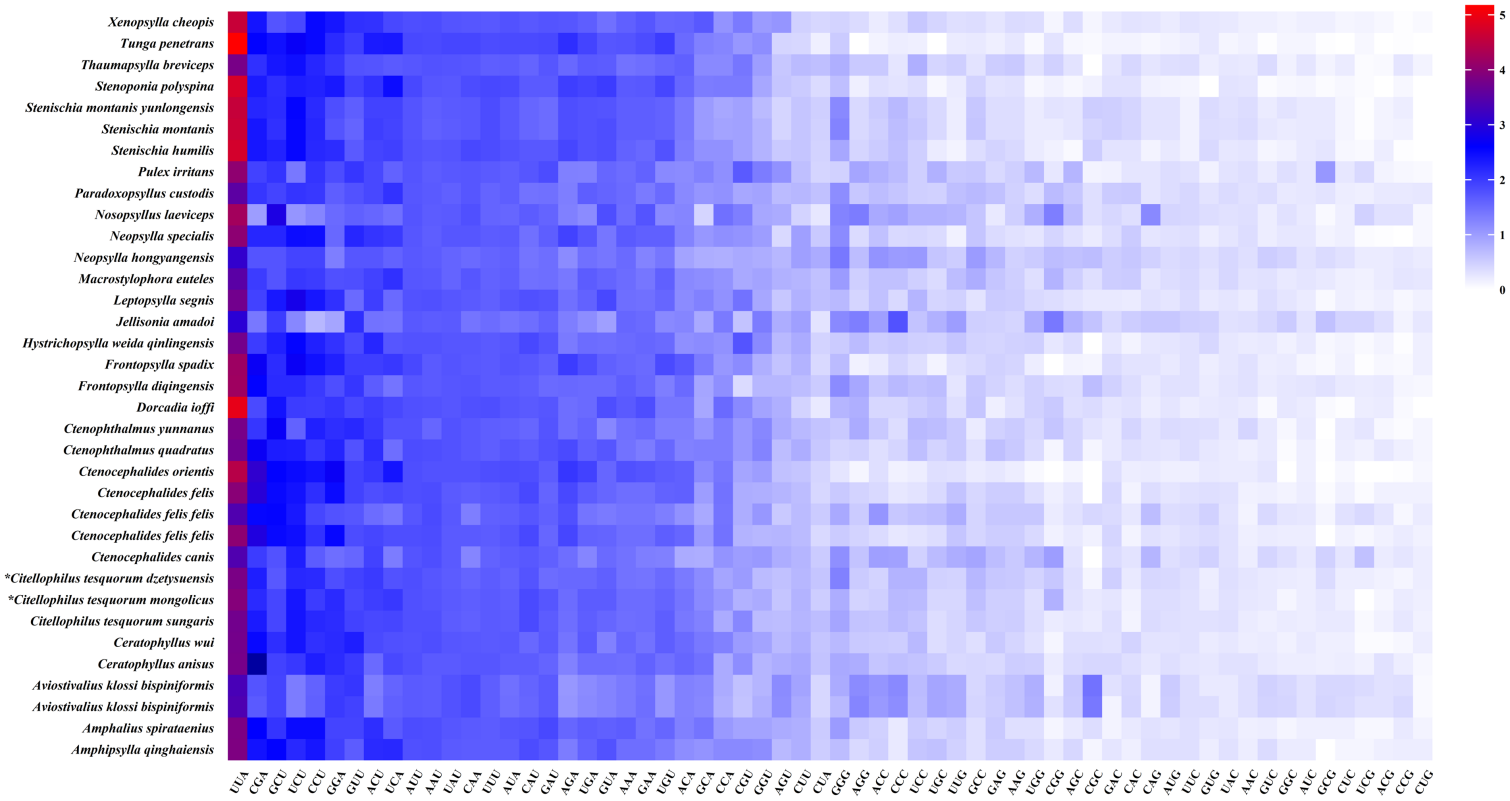
Relative synonymous codon usage (RSCU) in the mitogenomes of fleas, where * denotes the two species analyzed in this study.

**Figure 6:**
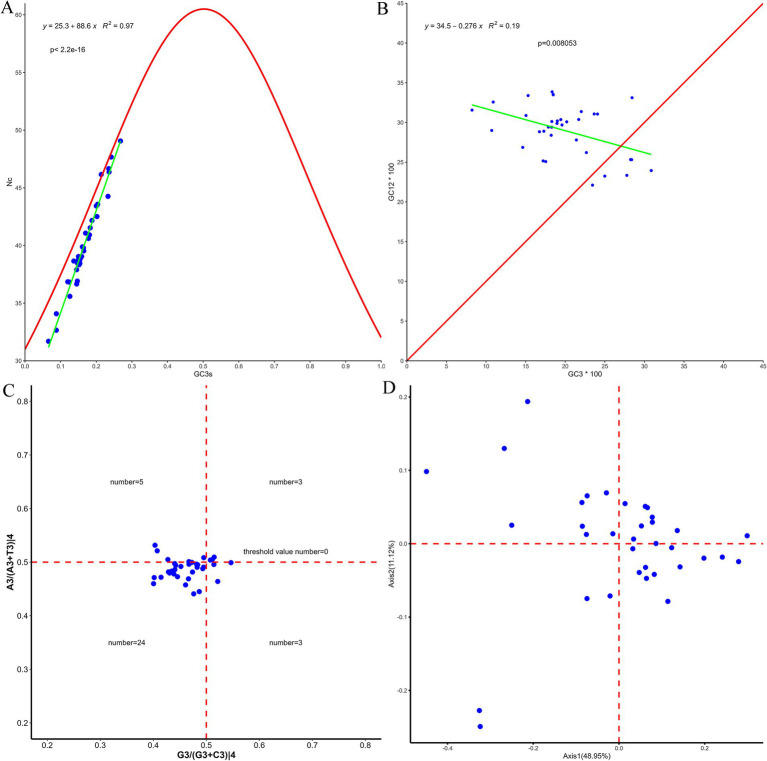
Analysis of 13 PCGs derived from 35 flea samples: **(A)** ENc-plot; **(B)** neutral plot; **(C)** PR2; **(D)** COA.

Statistical analysis of RSCU values revealed that the optimal codons in PCGs of the flea mitochondrial genome predominantly end in A or U. The most frequently used codons include UUA (Leu2), CGA (Arg), GCU (Ala), UCU (Ser2), and CCU (Pro), which collectively indicate a distinct AT bias in codon usage (Figure 5). The ENC values of 13 PCGs from 35 species of the order Siphonaptera ranged from 31.96 to 49.06, with an average exceeding 35, suggesting a weak codon usage bias. Except for *C. canis*, all other data points fell below the expected curve, suggesting that codon usage patterns were predominantly influenced by natural selection (Figure 6A). Neutral plot analysis revealed that GC12 values varied between 0.22 and 0.34, whereas GC3s values ranged from 0.08 to 0.31, with the slope of the regression curve being −0.276. Furthermore, no significant correlation was observed between GC12 and GC3s. These findings suggest that mutation pressure has a limited influence on the formation of codon bias, contributing only 27.6%, while other factors, particularly natural selection, appear to play a more prominent role in shaping codon usage patterns (Figure 6B). PR2 plot analysis indicated that codon usage bias is influenced by both natural selection and mutational pressure. The uneven distribution of data points across the four quadrants showed a predominant clustering in the lower left quadrant, with relatively fewer points in the other three quadrants. This distribution suggests a notable bias in the nucleotide composition at the third codon position, indicating a preference for T/C nucleotides (Figure 6C). In the COA analysis, the first four axes accounted for 48.95, 11.12, 9.04, and 7.31% of the variation in codon usage bias across species, respectively, with the first axis exhibiting the highest contribution. Most species were clustered closely together, while only a few were distinctly separated and dispersed at a distance (Figure 6D).

### 3.5 Transfer and ribosomal RNA genes

The 22 tRNA genes across all 35 mitogenomes varied in length from 54 bp (*trnH* in *Neopsylla specialis*) to 74 bp (*trnM* in *Nosopsyllus laeviceps*). The total length of tRNA sequences ranged from 1,405 bp in *Ctenophthalmus quadratus* and *Stenoponia polyspina* to 1,439 bp in *Frontopsylla spadix* and *Pulex irritans*. Specifically, the total lengths of tRNAs in *C. t. dzetysuensis* and *C. t. mongolicus* were 1,429 bp and 1,431 bp, respectively (Table 1). Among these 22 tRNAs genes, 14 are located on the H-strand, while the remaining eight are found on the L-strand. The AT content of tRNA genes across the 35 mitogenomes ranged from 78.1 to 81.1%. The AT skew varied between −0.009 and 0.047, while the GC skew exhibited a positive value ranging from 0.084 to 0.182 (Figure 2). Except for *trnS1* (tct), which lacks the DHU arm, the other 21 tRNAs of the two newly sequenced species exhibit typical cloverleaf structures. A total of 24 mismatches were identified in *C. t. dzetysuensis*, comprising 18 G-U, 3 U–U, 1 A-C, 1 A-G, and 1 A-A mismatch. In contrast, *C. t. mongolicus* displayed 25 G-U, 3 U–U, 1 C-C, 1 A-G, and 1 A-A mismatch, resulting in a total of 31 mismatches (Supplementary Figure S3).

The total length of the two rRNA genes across the 35 mitogenomes ranged from 1,970 bp in *N. laeviceps* to 2,109 bp in *Xenopsylla cheopis*. The overall AT content of these genes varied between 79.5 and 83.4%, with AT skew values ranging from −0.024 to 0.039 and positive GC skew values spanning from 0.298 to 0.376 (Figure 2). Both rRNA genes were conducted on the L strand, with the *rrnL* gene located between *trnL1* and *trnV*, exhibiting a length variation of 1,196 bp to 1,317 bp. In contrast, the *rrnS* gene displayed a length range of 774 bp in *N. laeviceps* to 799 bp in *C. orientis* (Table 1).

### 3.6 Phylogenetic analysis

Saturation analysis of nucleotide sequence base substitutions in the PCGs dataset, conducted using DAMBE, revealed that the Iss < Iss.c (*p* < 0.05), indicating the absence of substitution saturation and confirming the suitability of the dataset for subsequent phylogenetic analyses (Supplementary Figure S4).

**Figure 7:**
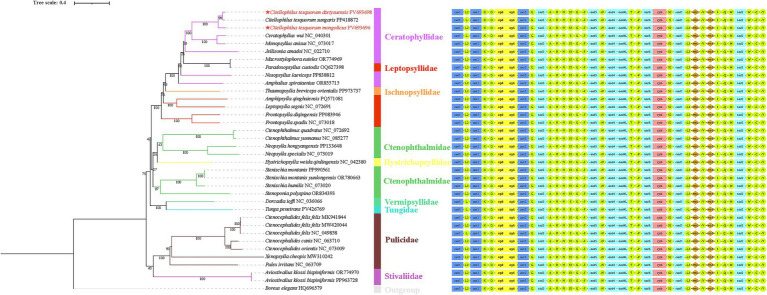
Phylogenetic trees were reconstructed using maximum likelihood (ML) method based on 13 PCGs from 35 species within the order Siphonaptera, with *Boreus elegans* (HQ696579) designated as the outgroup. Bootstrap support values are indicated at the nodes. The two species newly sequenced in this study are highlighted by red labels marked with an asterisk (★).

**Figure 8:**
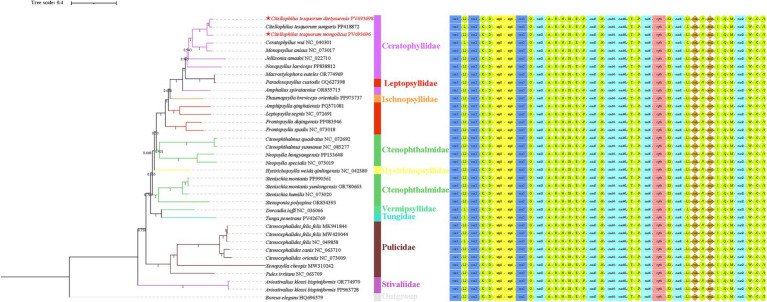
Phylogenetic trees were reconstructed using Bayesian inference (BI) method based on 13 PCGs from 35 species within the order Siphonaptera, with *Boreus elegans* (HQ696579) designated as the outgroup. Bayesian posterior probabilities are indicated at the nodes. The two species newly sequenced in this study are highlighted by red labels marked with an asterisk (★).

In this study, the nucleotide sequences of 13 PCGs from 35 species across 9 families within the order Siphonaptera, along with *Boreus elegans* (HQ696579) as an outgroup, were utilized to construct phylogenetic trees using both Maximum Likelihood (ML) and Bayesian Inference (BI) methods. The resulting trees exhibited identical topologies, with the exception of *N. laeviceps* (PP838812) and *Aviostivalius klossi bispiniformis* (OR774970, PP963728). In the ML tree, the 35 flea species were grouped into two major clades: the first clade consisted of [(Ceratophyllidae + Leptopsyllidae + Ischnopsyllidae) + (Ctenophthalmidae + Hystrichopsyllidae)] + (Vermipsyllidae + Tungidae), while the second clade included Pulicidae and Stivaliidae (Figure 7). In contrast, the BI tree revealed Stivaliidae as a distinct clade, with Pulicidae forming a sister group to the remaining families (Figure 8). Both ML and BI analyses supported the monophyly of Pulicidae (bp = 100, pp = 1). In contrast, Ceratophyllidae, Leptopsyllidae, and Ctenophthalmidae were found to be paraphyletic. The monophyly of the remaining families could not be confidently assessed due to the inclusion of only a single species in each. The two topological configurations indicated that *C. t. dzetysuensis* was most closely related to *C. t. sungaris* (bp = 99, pp = 1), with *C. t. mongolicus* forming a sister clade to this pair (bp = 100, pp = 1) (Figures 7, 8).

## 4 Discussion

This study aims to achieve the following objectives: (1) to accurately identify the target flea subspecies—*C. t. dzetysuensis* and *C. t. mongolicus*—based on diagnostic morphological characters prior to molecular analysis; (2) to assemble, annotate, and comparatively analyze their complete mitochondrial genomes; (3) to conduct a broad-scale comparative analysis of mitochondrial genome architecture and sequence variation across representative taxa within the order Siphonaptera; and (4) to reconstruct phylogenetic relationships among Siphonaptera. Integrating rigorous morphological identification with high-resolution mitochondrial genomic data enhances both specimen authentication and the inferential reliability of molecular phylogenetic conclusions.

Morphological identification represents the fundamental and essential preliminary step for integrative taxonomy and reliable molecular analysis. In this study, all flea specimens were accurately identified to the subspecies level based on diagnostic morphological characteristics, ensuring that only target individuals of *C. t. dzetysuensis* and *C. t. mongolicus* were selected for mitochondrial genome sequencing. Most existing diagnostic illustrations for fleas are hand-drawn and lack clear, high-resolution color photographs. In this study, we obtained clear morphological images of both male and female fleas, combining traditional morphological descriptions with intuitive photographic documentation to establish relatively complete and reliable morphological identification data.

In this study, the mitochondrial genomes of *C. t. dzetysuensis* and *C. t. mongolicus* were sequenced and comparatively analyzed with those of 33 other flea species retrieved from NCBI. The findings demonstrate that the mitogenome structure in fleas is highly conserved, retaining the ancestral gene order typical of insects (37), with no evidence of gene rearrangements detected across the examined taxa. Furthermore, the mitochondrial genomes and individual gene regions within Siphonaptera exhibit a pronounced AT bias in base composition—a characteristic commonly found in arthropods. This nucleotide preference is likely attributable to asymmetric mutational pressures and selective forces associated with replication and transcription processes (38). Animal mitochondrial tRNA genes typically exhibit a cloverleaf secondary structure, and the absence of a DHU arm in *trnS1* observed in the two species analyzed in this study is a well-documented characteristic of metazoan mitochondrial genome (36). The predominant mismatch detected in these tRNA genes is the G-U wobble pair. Accumulating evidence from previous studies indicates that G-U wobble pairs play a critical role in maintaining tRNA structural stability by preserving stem-region base pairing, which supports the overall cloverleaf conformation. Furthermore, these non-canonical pairings contribute to the structural specificity of mitochondrial tRNAs, ensuring their correct folding and functional integration into the mitochondrial translation (39).

The analysis of nucleotide diversity and evolutionary rate revealed that the *atp8* gene exhibited the highest values among 13 PCGs, indicating its potential as a molecular marker for studies on species evolution. In contrast, the *cox1* gene is highly conserved due to strong purifying selection, making it suitable for use as a DNA barcode in species identification. Codons ending in A or U were predominantly preferred, as evidenced by RSCU values exceeding 1, whereas those ending in G or C generally showed neutral usage with RSCU values below 1, a trend that consistent with codon usage trends observed across diverse metazoan species (40, 41). Results from RSCU and COA indicated minimal variation in codon usage among species within the order Siphonaptera. Furthermore, findings from the ENC-plot, neutral plot, and PR2 plot suggest that codon usage bias in the PCGs of the 35 Siphonaptera species is primarily shaped by natural selection, although other factors may also contribute.

Phylogenetic studies based on molecular markers using various single-gene combinations generally support the monophyly of Pulicidae, Ceratophyllidae, and Pygiopsyllidae, whereas Ctenophthalmidae is recovered as a paraphyletic group (42, 43). In the study by Whiting et al. (42), Leptopsyllidae was initially identified as a paraphyletic group; however, subsequent analyses incorporating additional *cox1* gene sequence data have clarified its monophyletic status (43). The findings of this study support the monophyly of Pulicidae, while Ceratophyllidae, Leptopsyllidae, and Ctenophthalmidae were found to be paraphyletic, consistent with most phylogenetic analyses based on mitochondrial genomes of fleas (44–47). Discrepancies in earlier studies may be attributed to limited taxon sampling, with fewer than 20 species included, thereby constraining the robustness of phylogenetic inference (48–50). Notably, Ctenophthalmidae was recovered as monophyletic in the phylogenetic tree constructed by Lin et al. (51) using PCGRNA-ML under codon-partitioning schemes. The systematic position of Hystrichopsyllidae remains unresolved: some studies support its paraphyly (52, 53), while others advocate for monophyly (54). Given that only a single representative species from this family was included in the present study, no definitive conclusion regarding its monophyletic status can be drawn.

The phylogenetic relationships recovered in this study provide important insights into the taxonomic status and evolutionary differentiation of *Citellophilus tesquorum* subspecies; both ML and BI trees showed that *C. t. dzetysuensi*s formed a strongly supported monophyletic clade with *C. t. sungaris*, while *C. t. mongolicus* occupied a sister position relative to this clade, indicating pronounced genetic divergence between *C. t. mongolicus* and the clade comprising *C. t. dzetysuensis* plus *C. t. sungaris*. This mitochondrial genomic divergence is consistent with previously reported genetic differentiation among *C. tesquorum* subspecies based on nuclear and mitochondrial markers (8), in which clear genetic isolation was detected with divergence levels approaching or exceeding those typically observed between closely related flea species, suggesting that some currently recognized subspecies may warrant re-evaluation as distinct valid species under integrated taxonomic frameworks. The observed phylogenetic structure may also reflect evolutionary differentiation linked to host association and geographic isolation, as *C. t. dzetysuensis* was collected from *Spermophilus undulatus* in Xinjiang and *C. t. mongolicus* from *Spermophilus dauricus* in Inner Mongolia, and their distinct hosts and allopatric distribution could reduce gene flow and drive adaptive divergence that together contributed to the mitogenomic divergence and the topological pattern of *C. t. mongolicus*. These results highlight that mitochondrial genomic data offer high resolution for evaluating species boundaries and subspecies validity in Siphonaptera, and future studies incorporating population sampling, nuclear markers, and morphological morphometrics will help clarify whether the observed divergence represents intraspecific variation or interspecific differentiation and further refine the taxonomic status of *C. tesquorum* subspecies under an integrative taxonomy framework.

This study establishes a novel molecular foundation for phylogenetic analysis and species identification within fleas. Nevertheless, certain limitations remain. First, the taxonomic sampling in this study is insufficient. Future efforts should focus on expanding the representation of flea taxa through broader sampling and additional mitochondrial genome sequencing to strengthen the robustness of phylogenetic inferences. Second, although mitogenomes are widely utilized in molecular phylogenetic and species identification due to their high resolution, integrating nuclear genomic data provides a critical means of addressing potential phylogenetic biases, thereby enabling a more accurate and comprehensive reconstruction of evolutionary relationships. Consequently, future studies should prioritize integrative multi-omics analyses to systematically refine and robustly support the phylogenetic framework of Siphonaptera.

## 5 Conclusion

This study newly sequenced and annotated the complete mitochondrial genomes of *C. t. dzetysuensis* and *C. t. mongolicus*. Morphological identification ensured accurate specimen sourcing, comparative mitogenomic analysis revealed high structural conservation across Siphonaptera. Phylogenetic analysis showed pronounced genetic divergence between the two subspecies, consistent with their current taxonomic status and suggestive of considerable evolutionary separation. The observed differentiation is likely associated with geographic isolation and host adaptation. This study improves the phylogenetic resolution of fleas and provides valuable molecular evidence for the taxonomy, evolution, and surveillance of plague vector fleas. Further studies including population sampling and nuclear markers will help to more precisely define species boundaries within the *Citellophilus tesquorum* species complex.

## Data Availability

The mitochondrial genome sequences of *C. t. dzetysuensis* and *C. t. mongolicus* have been deposited in the National Center for Biotechnology Information (NCBI) database (https://www.ncbi.nlm.nih.gov/) under accession numbers PV693698 and PV693696, respectively.

## Footnotes

1
https://github.com/rrwick/Bandage

2
http://mitos.bioinf.uni-leipzig.de/

3
https://sourceforge.net/projects/codonw/
