# Supplementary material for: Mitochondrial genomes of two subspecies of Citellophilus tesquorum: Insights into the molecular evolution and phylogenetic analysis within Siphonaptera
Source: Front Vet Sci. 2026 Apr 29;13:1785674. doi: 10.3389/fvets.2026.1785674 (PMC13168148; doi:10.3389/fvets.2026.1785674)
Supplement: Supplementary file 1 [file Data_Sheet_1.docx]

| Datasets | Subset partitions | Best model |
| --- | --- | --- |
| PCG(ML) | P1: (atp6_codon1, cox1_codon1, cox2_codon1, cox3_codon1, cytb_codon1) | GTR+F+I+I+R3 |
|  | P2: (atp6_codon2, cox1_codon2, cox2_codon2, cox3_codon2, cytb_codon2, nad1_codon2, nad2_codon2, nad3_codon2, nad4L_codon2, nad4_codon2, nad5_codon2, nad6_codon2) | GTR+F+I+I+R4 |
|  | P3: (atp6_codon3, atp8_codon3, cox1_codon3, cox3_codon3, cytb_codon3, nad3_codon3, nad6_codon3) | TIM2+F+R6 |
|  | P4: (atp8_codon1, nad1_codon3, nad4_codon3) | TIM3+F+I+I+R3 |
|  | P5: (atp8_codon2, nad2_codon1, nad3_codon1, nad6_codon1) | TIM2+F+I+G4 |
|  | P6: (cox2_codon3, nad2_codon3) | HKY+F+G4 |
|  | P7: (nad1_codon1, nad4L_codon1, nad4_codon1, nad5_codon1) | TVM+F+I+G4 |
|  | P8: (nad4L_codon3, nad5_codon3) | TIM3+F+I+I+R3 |
|  |  |  |
| PCG(BI) | P1: (atp6_codon1, cytb_codon1) | GTR+F+I+G4 |
|  | P2: (atp6_codon2, cox1_codon2, cox2_codon2, cox3_codon2, cytb_codon2) | GTR+F+I+G4 |
|  | P3: (atp6_codon3, atp8_codon3, cox2_codon3, nad2_codon3, nad3_codon3, nad6_codon3) | HKY+F+I+G4 |
|  | P4: (atp8_codon1) | HKY+F+I+G4 |
|  | P5: (atp8_codon2, nad2_codon1, nad3_codon1, nad6_codon1) | GTR+F+I+G4 |
|  | P6: (cox1_codon1, cox2_codon1, cox3_codon1) | GTR+F+I+G4 |
|  | P7: (cox1_codon3) | HKY+F+G4 |
|  | P8: (cox3_codon3, cytb_codon3) | HKY+F+G4 |
|  | P9: (nad1_codon1, nad4L_codon1, nad4_codon1, nad5_codon1) | GTR+F+I+G4 |
|  | P10: (nad1_codon2, nad2_codon2, nad3_codon2, nad4L_codon2, nad4_codon2, nad5_codon2) | GTR+F+I+G4 |
|  | P11: (nad1_codon3, nad4L_codon3, nad4_codon3, nad5_codon3) | GTR+F+I+G4 |
|  | P12: (nad6_codon2) | GTR+F+G4 |

**Table S1** Optimal nucleotide substitution models for constructing maximum likelihood and Bayesian trees with the PCGs datasets.

**Table S2** Organization of the mitochondrial genome of *Citellophilus tesquorum dzetysuensis* and *Citellophilus tesquorum mongolicus*.

| Feature | Strand | Position(start-end) | Length(bp) | Initiation_codon | Stop_codon | Anticodon | Intergenicnucleotide |
| --- | --- | --- | --- | --- | --- | --- | --- |
| *trnI* | H | 1-64/337-400 | 64/64 |  |  | GAT | 1/1 |
| *trnQ* | L | 66-134/402-470 | 69/69 |  |  | TTG | -1/14 |
| *trnM* | H | 134-201/485-552 | 68/68 |  |  | CAT | 0/15 |
| *nad2* | H | 202-1,215/568-1,569 | 1,014/1002 | ATT/ATG | TAA/TAA |  | -1/0 |
| *trnW* | H | 1,215-1,280/1,570-1,634 | 66/65 |  |  | TCA | 37/6 |
| *trnC* | L | 1,318-1,378/1,641-1,701 | 61/61 |  |  | GCA | 0/0 |
| *trnY* | L | 1,379-1,442/1,702-1,765 | 64/64 |  |  | GTA | -3/-3 |
| *cox1* | H | 1,440-2,975/1,763-3,298 | 1,536/1,536 | ATC/ATC | TAA/TAA |  | 4/4 |
| *trnL2* | H | 2,980-3,043/3,303-3,366 | 64/64 |  |  | TAA | 1/1 |
| *cox2* | H | 3,045-3,725/3,368-4,048 | 681/681 | ATG/ATG | TAA/TAA |  | 2/2 |
| *trnK* | H | 3,728-3,797/4,051-4,120 | 70/70 |  |  | CTT | 0/0 |
| *trnD* | H | 3,798-3,862/4,121-4,185 | 65/65 |  |  | GTC | 0/9 |
| *atp8* | H | 3,863-4,024/4,195-4,347 | 162/153 | ATT/ATA | TAA/TAA |  | -7/-7 |
| *atp6* | H | 4,018-4,692/4,341-5,015 | 675/675 | ATG/ATG | TAA/TAA |  | -1/-1 |
| *cox3* | H | 4,692-5,474/5,015-5,797 | 783/783 | ATG/ATG | TAA/TAA |  | 0/0 |
| *trnG* | H | 5,475-5,538/5,798-5,860 | 64/63 |  |  | TCC | 62/62 |
| *nad3* | H | 5,601-5,951/5,923-6,273 | 351/351 | ATC/ATT | TAG/TAG |  | -2/-2 |
| *trnA* | H | 5,950-6,012/6,272-6,334 | 63/63 |  |  | TGC | 2/3 |
| *trnR* | H | 6,015-6,076/6,338-6,399 | 62/62 |  |  | TCG | -3/-3 |
| *trnN* | H | 6,074-6,139/6,397-6,462 | 66/66 |  |  | GTT | 0/0 |
| *trnS1* | H | 6,140-6,207/6,463-6,530 | 68/68 |  |  | TCT | 0/0 |
| *trnE* | H | 6,208-6,271/6,531-6,595 | 64/65 |  |  | TTC | -2/-2 |
| *trnF* | L | 6,270-6,334/6,594-6,658 | 65/65 |  |  | GAA | 0/9 |
| *nad5* | L | 6,335-8,051/6,668-8,375 | 1,717/1,708 | ATG/ATG | T/T |  | 1/1 |
| *trnH* | L | 8,053-8,115/8,377-8,439 | 63/63 |  |  | GTG | 0/0 |
| *nad4* | L | 8,116-9,451/8,440-9,709 | 1,336/1,270 | ATG/ATG | T/T |  | -7/59 |
| *nad4l* | L | 9,445-9,738/9,769-10,062 | 294/294 | ATG/ATG | TAA/TAA |  | 2/2 |
| *trnT* | H | 9,741-9,804/10,065-10,128 | 64/64 |  |  | TGT | 0/0 |
| *trnP* | L | 9,805-9,868/10,129-10,195 | 64/67 |  |  | TGG | 11/11 |
| *nad6* | H | 9,880-10,386/10,207-10,713 | 507/507 | ATT/ATT | TAA/TAA |  | -1/-1 |
| *cob* | H | 10,386-11,525/10,713-11,852 | 1,140/1,140 | ATG/ATG | TAA/TAA |  | 6/2 |
| *trnS2* | H | 11,532-11,596/11,855-11,919 | 65/65 |  |  | TGA | 16/22 |
| *nad1* | L | 11,613-12,554/11,942-12,883 | 942/942 | ATG/ATG | TAA/TAA |  | 1/1 |
| *trnL1* | L | 12,556-12,617/12,885-12,946 | 62/62 |  |  | TAG | 0/0 |
| *rrnL* | L | 12,618-13,912/12,947-14,242 | 1,295/1,296 |  |  |  | 0/0 |
| *trnV* | L | 13,913-13,980/14,243-14,310 | 68/68 |  |  | TAC | 0/0 |
| *rrnS* | L | 13,981-14,760/14,311-15,090 | 780/780 |  |  |  | 0/0 |
| OH | H | 14,761-16,458/15,091-15,373 | 1,698/283 |  |  |  | 0/-1 |


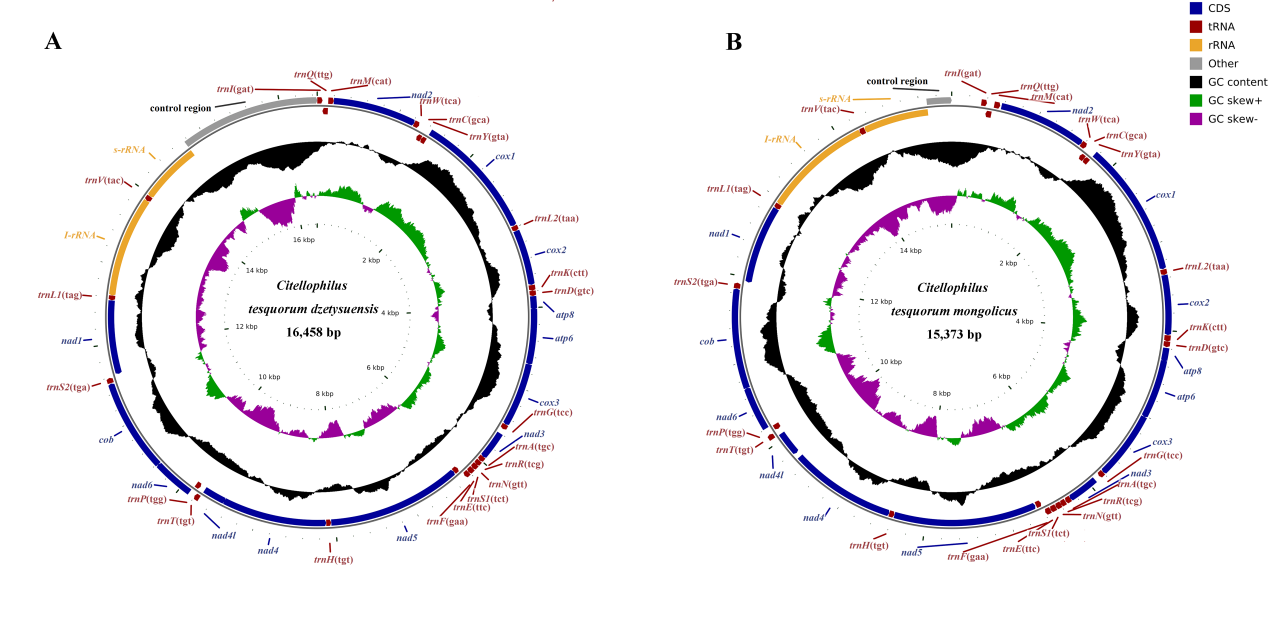


**Fig.S1.** The complete mitogenome maps of *Citellophilus tesquorum dzetysuensis* (A) and *Citellophilus tesquorum mongolicus* (B).


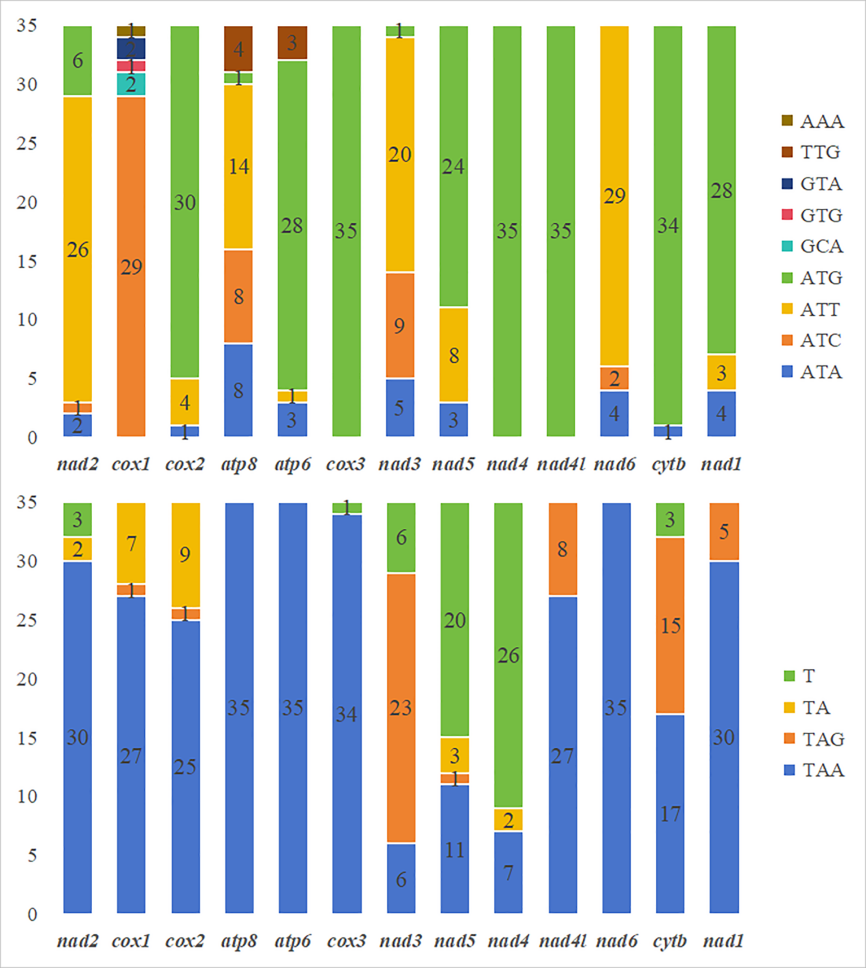


**Fig.S2.** The usage of start codons (A) and stop codons (B) for mitohondrial protein-coding genes in the order Siphonaptera.

**
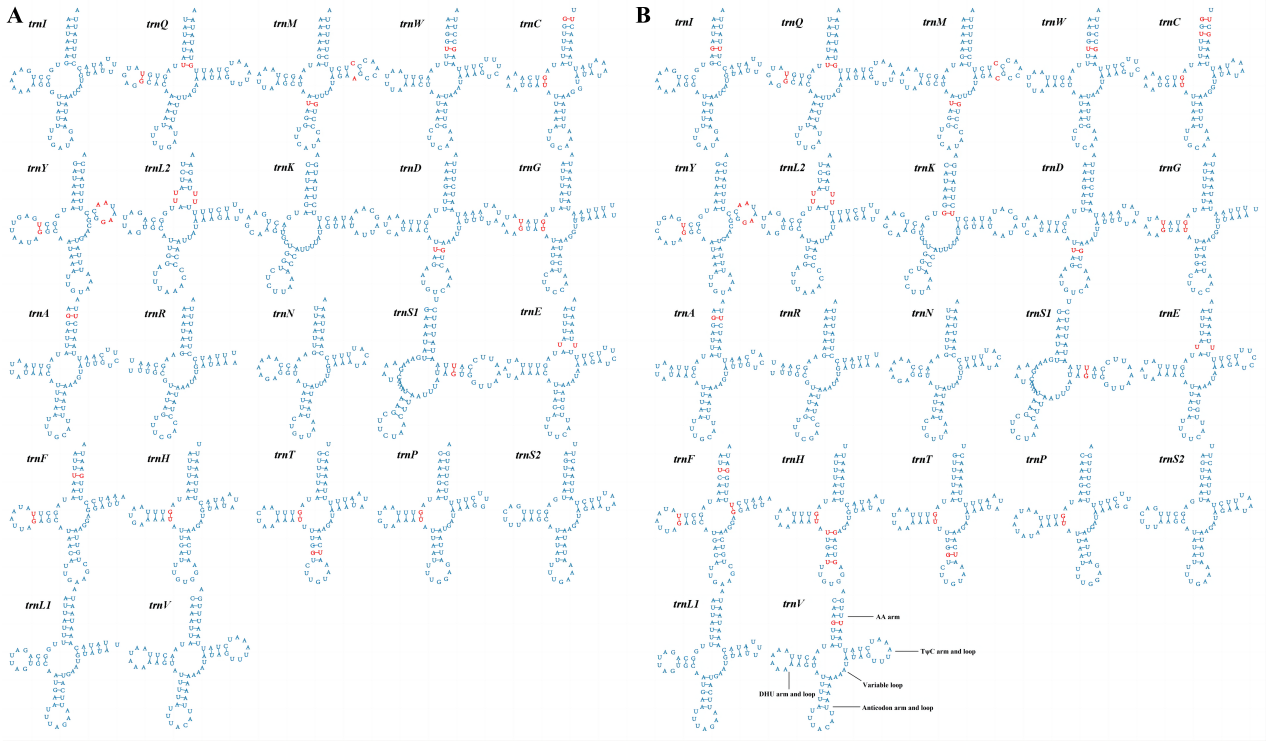
**

**Fig.S3.** Secondary structure of the 22 mitochondrial tRNA genes of *Citellophilus tesquorum dzetysuensis* (A) and *Citellophilus tesquorum mongolicus* (B).

**
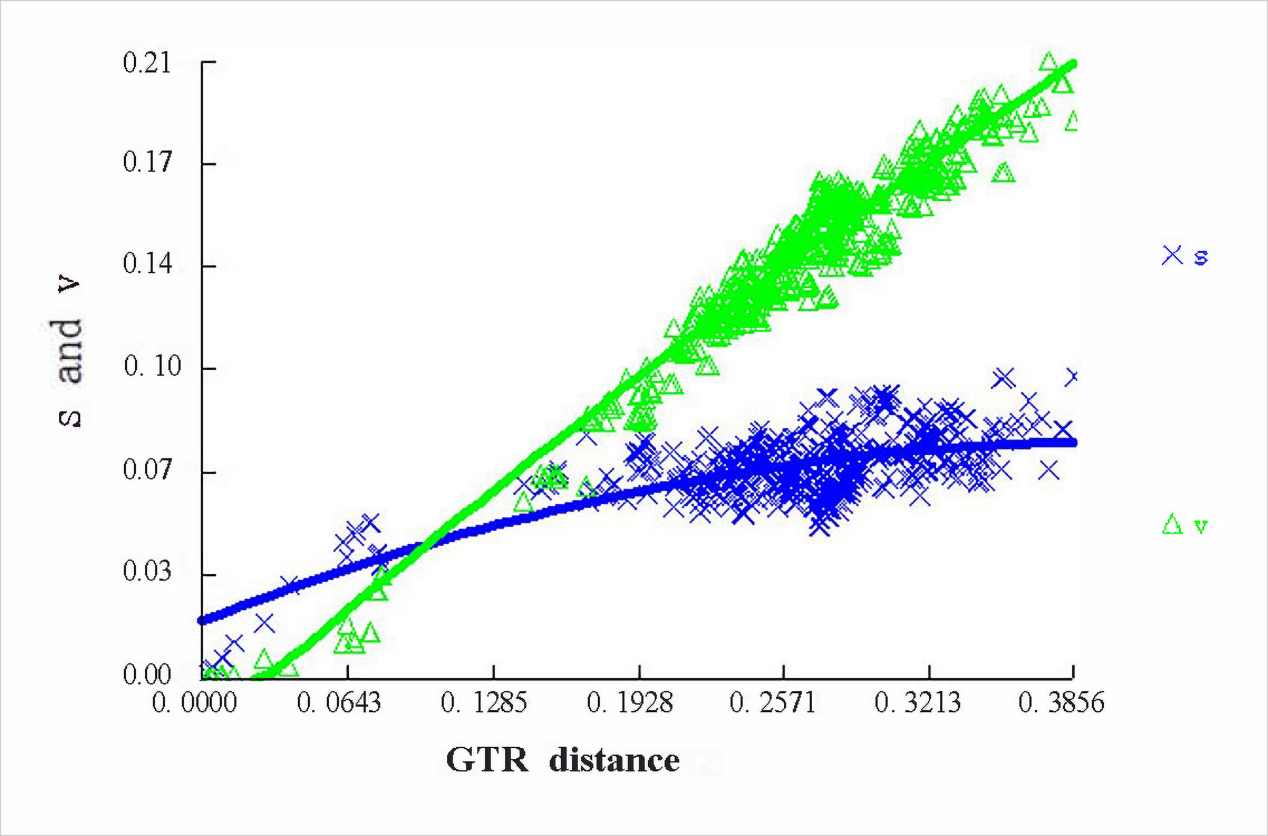
**

**Fig.S4.** Base substitution saturation analysis of 13 PCGs.
